# Epidemiology of *Burkholderia cepacia* Complex in Patients with Cystic Fibrosis, Canada

**DOI:** 10.3201/eid0802.010163

**Published:** 2002-02

**Authors:** David P. Speert, Deborah Henry, Peter Vandamme, Mary Corey, Eshwar Mahenthiralingam

**Affiliations:** * University of British Columbia and Children’s and Women’s Health Centre of British Columbia, Vancouver, British Columbia, Canada; † University of Ghent, Ghent, Belgium; ‡ The Hospital for Sick Children, Toronto, Ontario, Canada

**Keywords:** *Burkholderia cepacia* complex, cystic fibrosis, epidemiology, genomovar, Canada

## Abstract

The *Burkholderia cepacia* complex is an important group of pathogens in patients with cystic fibrosis (CF). Although evidence for patient-to-patient spread is clear, microbial factors facilitating transmission are poorly understood. To identify microbial clones with enhanced transmissibility, we evaluated *B. cepacia* complex isolates from patients with CF from throughout Canada. A total of 905 isolates from the *B. cepacia* complex were recovered from 447 patients in 8 of the 10 provinces; 369 (83%) of these patients had genomovar III and 43 (9.6%) had *B. multivorans* (genomovar II). Infection prevalence differed substantially by region (22% of patients in Ontario vs. 5% in Quebec). Results of typing by random amplified polymorphic DNA analysis or pulsed-field gel electrophoresis indicated that strains of *B. cepacia* complex from genomovar III are the most potentially transmissible and that the *B. cepacia* epidemic strain marker is a robust marker for transmissibility.

*Burkholderia cepacia* complex is an important group of pathogens in immunocompromised hosts, notably those with cystic fibrosis (CF) or chronic granulomatous disease (1,2). Lung infections with *B. cepacia* complex in certain patients with CF result in rapidly progressive, invasive, fatal bacteremic disease (3). Furthermore, the bacteria have a potential for patient-to-patient spread, both within and outside the hospital (4–9), raising questions about optimal measures for infection control.

The disease risk for infection with *B. cepacia* complex in patients with CF is substantially higher than with *Pseudomonas aeruginosa* alone or with bacteria other than *B. cepacia* or *P. aeruginosa* (10). However, there is a dramatic heterogeneity in outcome among CF patients infected with *B. cepacia* complex: some patients have a fulminant decline in pulmonary function, and others harbor *B. cepacia* complex for extended periods of time with no obvious adverse effects. The marked difference in prognosis among infected patients has not been adequately explained but is thought to result in part from differences among infecting strains of *B. cepacia* complex.

*B. cepacia* is a genetically highly diverse class of bacteria, which is composed of several different species and discrete groups constituting the *B. cepacia* complex (11). Each group differs sufficiently from the others to constitute a species, and those that are phenotypically distinct have been assigned species designation. Those that cannot be differentiated phenotypically but are genetically distinct are defined as genomovars (11). As phenotypic differentiation among the genomovars has improved over the past decade, new species designation has been assigned as follows: genomovar II = *B. multivorans*, genomovar IV = *B. stabilis*, genomovar V = *B. vietnamiensis*, and genomovar VII = *B. ambifaria*. Genomovars I and III cannot be differentiated phenotypically, nor can *B. multivorans* and genomovar VI; these species must be distinguished by genetic methods. Bacteria from each of the genomovars have been recovered from patients with CF, but the predominant isolates in North America are from genomovar III and *B. multivorans* (12).

Numerous questions about the epidemiology of *B. cepacia* complex in CF are unanswered; for example, it is not known if certain genomovars or strains are more virulent than others. The relative risk for patient-to-patient spread of strains from each of the different genomovars is also unknown. Two genetic elements have been identified in strains having a propensity for epidemic spread. First, *cblA,* which encodes the protein for cable pilus production, is found in a single highly transmissible lineage from genomovar III that clusters among patients in the United Kingdom and Canada (13). Second, the B. cepacia epidemic strain marker (BCESM), which encodes a protein of unknown function, is found in many different strains from genomovar III, each of which is clustered in specific CF treatment centers (14).

Infection with bacteria from the *B. cepacia* complex has a profound effect on the lives of patients with CF. Since *B. cepacia* complex infection can be spread from one CF patient to another, provisions have been introduced in hospitals to limit contact among these patients. Infected patients are prohibited in some countries from attending social gatherings where other CF patients may be in attendance. Furthermore, since virulence appears to differ among strains and one strain may replace another, policies have been introduced in some centers to limit contact among patients who are infected with any strain from the *B. cepacia* complex. Lack of a clear understanding about the epidemiology of *B. cepacia* complex and the relative risk of infection with each of the different genomovars has spawned anxiety and confusion among CF patients, their caregivers, and families. Infection control policies have been developed in an effort to balance the rights of CF patients with careful consideration of their physical and mental health.

With burgeoning knowledge about the taxonomy, epidemiology, and virulence of the *B. cepacia* complex, many questions about appropriate infection control precautions have been raised. Consensus has been difficult to attain because of incomplete and conflicting data from various regions throughout the world. We undertook this study to provide a database from which infection control questions could begin to be answered.

In 1994 a *B. cepacia* complex research and referral repository for Canadian CF clinics was established in Vancouver. Since that time, *B. cepacia* complex isolates from an estimated 75% of infected Canadian CF patients have been evaluated for genomovar and species identity, random amplified polymorphic DNA (RAPD) strain type, and putative markers of transmissibility. These data have permitted inferences about the potential transmissibility of different strains and facilitated the development of rational infection control guidelines. We report data and conclusions from our observations to date.

## Materials and Methods

### Patients and Clinics

Canadian CF clinics are linked through the Canadian Cystic Fibrosis Foundation, which collates annual summary data in its patient data registry. Approximately 3,200 patients with CF receive care at 36 clinics in the 10 provinces. Each clinic provides care for 20 to 300 patients (median age 17 years). The number of patients with CF in Canada has increased by approximately 90 each year since 1994, although the median survival age has plateaued at approximately 30 years.

In 1994, the Canadian *B. cepacia* Complex Research and Referral Repository was established at the British Columbia Research Institute for Children’s and Women’s Health in Vancouver. Each clinic director was notified about the new laboratory and encouraged to send archived and new isolates of *B. cepacia* complex to the Vancouver laboratory for strain typing and confirmation of species identity (7). At least one isolate from each infected patient was solicited, as well as subsequent isolates that were considered phenotypically different.

### Species and Genomovar Determination

A polyphasic scheme (15) was used to determine the species or genomovar classification of each isolate.

### Phenotypic Identification of *B. cepacia* Complex and Other Organisms

Isolates were identified as described (15,16): purity, morphology, and hemolysis were observed, and oxidase activity (Pathotec cytochrome oxidase, Remel, Lenexa, KA) was tested after growth on Columbia agar with 5% sheep blood (PML Microbiologicals, Richmond, British Columbia, Canada). Bacteria were incubated for up to 7 days at 35°C in the following sugars: glucose, maltose, lactose, xylose, sucrose, and adonitol. Moeller lysine, ornithine, and negative control were also heavily inoculated and incubated at 35°C for 48 hours. The API 20 NE strip (Biomerieux Vitek Inc., Hazelwood, MO) was set up according to manufacturer’s instructions, except that the strip was incubated at 35°C and observed at 24 and 48 hours. Growth on MacConkey agar without crystal violet (Difco Laboratories, Detroit, MI) and on *Burkholderia cepacia* selective agent (15) at 35°C was observed at 24 and 48 hours. Pigment production and growth on tryptic soy agar at 35°C and 42ΕC were observed at 24 and 48 hours.

## Molecular Methods

### Genomovar-Specific PCR for the *recA* Gene

Polymerase chain reaction (PCR) with selected *recA* primers was performed essentially as described (16,17). Tests were done with the six *recA* subgroup genomovar-specific primers (16,17) and a seventh primer pair for genomovar VII as described (16,18). After amplification, 8 µL of each reaction mixture was subjected to electrophoresis in 1.5% agarose gel. PCR products were photographed after ethidium bromide staining. *B. cepacia* complex strains that did not react with the specific primers described above were subjected to nucleotide sequence analysis of the *recA* gene (17). Placement in the complex was then done phylogenetically by analysis of 500 bp of the N-terminal encoding sequence according to the algorithm described (17).

### Speciation with the 16S *rRNA* Gene

Restriction fragment length polymorphism (RFLP) analysis of the 16S *rRNA* gene PCR product with the enzyme *Dde*1 was performed as described (16,17).

### Genotypic Identification of *B. gladioli*

A PCR reaction with primer pair LP1/LP4, directed toward a species-specific region of the 23S *rRNA* gene, was used (19).

### Strain Typing of *B. cepacia* Complex Isolates

Each isolate was evaluated for RAPD strain type by RAPD analysis (20). If typing results were ambiguous, pulsed-field gel electrophoresis (PFGE) analysis was performed (21).

Groups of isolates that were unambiguously identical by RAPD with or without PFGE were each assigned numerical types. Unique isolates were designated X until another identical isolate was identified.

### Evaluation for Markers of Transmissibility

Southern dot blot analysis was performed (14) to determine if each isolate encoded either of the genetic markers of transmissibility (*cblA* or BCESM).

## Results

### Isolates Received from Canadian CF Clinics

A total of 922 isolates considered to be *B. cepacia* complex (or possible *B. cepacia* complex) by the referring laboratory were received through July 2000 (Table 1). These isolates were recovered from 459 different patients. Most specimens were received after a request for strains was made in 1994 through the Canadian Cystic Fibrosis Foundation; however, 95 isolates were archived specimens obtained from 1981 to 1991. Seventeen isolates (from 17 patients) were organisms that had been misidentified as *B. cepacia* complex. Fourteen of these 17 isolates had been received before 1997, when information about the importance of correct identification began to be disseminated by the International *B. cepacia* Working Group (http://allserv.rug.ac.be/~tcoenye/). The 17 isolates included 5 of *Stenotrophomonas maltophilia*; 4 of *Pseudomonas* species; 3 of *Alcaligenes xylosoxidans*; and 1 each of *Enterobacter agglomerans*, *Candida* species, mixed gram-positive bacteria, and 2 undescribed new species. Eighteen isolates belonged to species that are phenotypically similar to *B. cepacia* complex (Table 2). *Pandoraea* species and *B. fungorum* have only recently been described (22,23).

**Table 1:** *Burkholderia cepacia* complex isolates and phenotypically similar organisms recovered from patients with cystic fibrosis in Canada

Province	No. of patients from whom isolates were submitted	Total no. of isolates submitted	No. of genomovar/species submitted from different patients		
			*B. multivorans* (genomovar II)	III	Other
British Columbia	95	394	33	52	20
Alberta	45	55	2	32	13
Manitoba	7	7	3	3	1
Saskatchewan	0	0	0	0	0
Ontario	243	292	2	233	8
Quebec	23	48	3	15	5
Prince Edward Island	0	0	0	0	0
New Brunswick	4	4	0	3	1
Nova Scotia	17	89	0	18	2
Newfoundland	13	16	0	13	0
Territories	0	0	0	0	0
Total (including territories)	447	905	43	369	50

**Table 2:** Genomovar or species of *Burkholderia cepacia* complex or phenotypically similar isolates from cystic fibrosis patients in Canada

Species or genomovar	No. of patients infected with species or genomovar^a^	Percentage of patients (%)
Genomovar I	1	0.2
*Burkholderia multivorans* (genomovar II)	43	9.3
Genomovar III	369	80.0
*Burkholderia stabilis* (genomovar IV)	17	3.8
*Burkholderia vietnamiensis* (genomovar V)	7	1.6
*Burkholderia cepacia* complex (not genomovar I-VII)	8	1.8
*Burkholderia fungorum*	1	0.2
*Burkholderia gladioli*	5	1.1
*Ralstonia pickettii*	5	1.1
*Pandoraea* spp.	5	1.1
Total	461	

^a^Some patients were counted twice if two or more different strains were recovered.

### Geographic Distribution of CF Patients Infected with *B. cepacia* Complex

Since this study was conducted by passive ascertainment of bacterial cultures, the representation from regions of Canada differed considerably. Isolates confirmed as members of the *B. cepacia* complex were received from 8 of 10 provinces (Table 1). Only Saskatchewan, Prince Edward Island, and the territories (Yukon, Nunavut, and Northwest) did not submit specimens.

The consistently monitored data in the Patient Data Registry of the Canadian Cystic Fibrosis Foundation provide good estimates of regional patterns of *B. cepacia* complex prevalence and repository representation. To provide more stable estimates and preserve confidentiality in provinces with a small number of centers, the cumulative prevalence of infection from 1992 to 1997 is reported for four regions of Canada (Table 3). The prevalence of *B. cepacia* complex was highest in Ontario and in the eastern provinces. Although Quebec and Ontario are contiguous and have similar populations, the prevalence of infection with *B. cepacia* complex was substantially higher in Ontario. The provinces west of Ontario had a combined prevalence intermediate between those of Ontario and the eastern provinces, but this region covers a very large geographic area, which may include heterogeneous provincial prevalence rates.

**Table 3:** Regional prevalence of cystic fibrosis (CF) patients infected with *Burkholderia cepacia* complex and representation of their isolates in Canadian *B. cepacia* Research and Referral Repository, 1992-1997

Region of Canada	1996 census, population, thousands	No. of CF patients, 1992-1997	No. (%) of CF patients infected with *B. cepacia* complex	No. (%) of CF patients with samples of *B. cepacia* complex in repository
West	8,816	975	117 (12)	91 (78)^a^
Ontario	11,101	1293	285 (22)	241 (85)
Quebec	7,274	1088	55 (5)	16 (29)
East	2,381	405	103 (25)	27 (26)

^a^Archived samples from the main study center have been excluded here to provide comparative regional estimates.

Although the relative prevalence of different genomovars varied from province to province, genomovar III was found in every province in which patients were infected with *B. cepacia* complex (Table 1). The only province from which *B. multivorans* was frequently recovered was British Columbia. Each isolate of *B. multivorans* was unique except for those recovered from sibling pairs (24).

### Prevalence of Genomovars and Species of Isolates from the *B. cepacia* Complex

All initial isolates and those that appeared phenotypically different from previously received isolates from individual patients were evaluated for genomovar and species identity by a polyphasic scheme involving both biochemical analyses and molecular methods (*recA* PCR and 16s *rRNA* RFLP) (Table 2). Most isolates (80%) were from genomovar III and included all strains that clustered in individual centers and appeared to be transmitted from patient to patient. Approximately 9% of infected patients were infected with *B. multivorans* (genomovar II), but there was little evidence among these isolates of genotypic clustering as determined by RAPD and PFGE. Isolates from the other genomovars and species (genomovar I, *B. stabilis,* *B. vietnamiensis,* and *B. cepacia* complex bacteria of indeterminate genomovar status) were recovered, but at very low frequency (Table 2). Several patients were infected with more than one strain or genomovar from the *B. cepacia* complex, but in all but one case, one strain replaced another that had been identified previously**.** Replacement of genomovar II (*B. multivorans*) by genomovar III occurred in six patients (24).

Eight patients were infected with *B. cepacia* complex bacteria that did not belong to any of the currently defined genomovars (Table 2). The full-length *recA* gene was amplified from these isolates by using primers BCR1 and BCR2 (17). These strains produced novel *recA* RFLP products, and none reacted with the PCR primers developed to identify the current genomovars (17,18). Their biochemical profile was consistent with that of the *B. cepacia* complex (data not shown). Analysis of the 16S rRNA gene by RFLP demonstrated that these strains were not *B. multivorans, B. vietnamiensis*, or genomovar VI, since they had the single RFLP profile shared by all the remaining current genomovars/species (*B. stabilis,* I, III, and VII; pattern 2 [17]). The nucleotide sequence of the *recA* gene from five isolates representative of these novel strains was examined phylogenetically (Figure). These strains form two unique, distinct clusters with the current *B. cepacia* complex (Figure), suggesting that they are members of the current complex but may be novel taxonomic groups or subgroups of the existing genomovars not detected by the current molecular tests.

**Figure:**
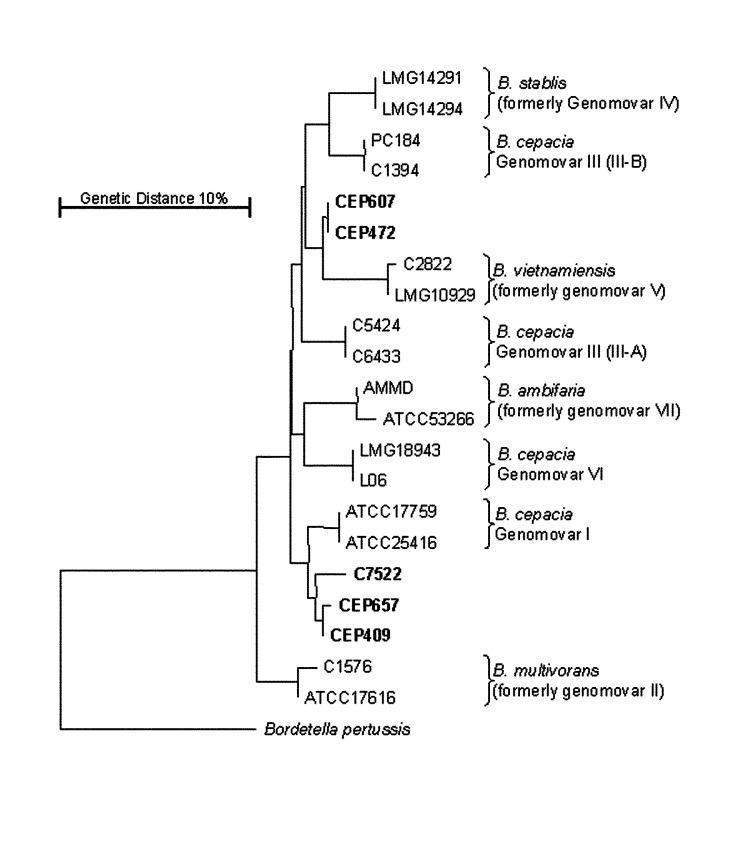
Phylogenetic analysis of the *recA* gene from the *Burkholderia cepacia* complex. The phylogenetic diversity of the *B. cepacia* complex observed after nucleotide sequence analysis of the *recA* gene is shown. Isolates recovered from Canadian CF patients that are representative of strains of currently indeterminate genomovar status (Table 2) appear in bold and lack species identification; all fall within the current *B. cepacia* complex. The tree was drawn as described (16). The *recA* sequence from *Bordetella pertussis* was used as a root, and the genetic distance is indicated by the bar.

### Geographic Distribution of *B. cepacia* Complex from Different Epidemic RAPD Strain Types

We have identified four genetic lineages of *B. cepacia* complex genomovar III that cluster by geographic region in Canada (Table 4). Each of these types was defined by RAPD and confirmed by PFGE. All the isolates from types 01, 04, and 06 harbored the BCESM, but only those from RAPD type 02 encoded both BCESM and *cblA*.

**Table 4:** Number of cystic fibrosis patients infected with *Burkholderia cepacia* complex genomovar III RAPD strain type, by Canadian province

Province	RAPD strain type				
	01	02	04	06	Other (BCESM+)^a^
British Columbia	9	11	16	7	10 (5)
Alberta	2	14	16	0	0
Manitoba	0	1	1	0	1 (0)
Saskatchewan	0	0	0	0	0
Ontario	1	223	2	0	7 (5)
Quebec	5	7	1	0	2 (2)
Prince Edward Island	0	0	0	0	0
New Brunswick	0	0	2	0	1 (0)
Nova Scotia	0	2	12	0	4 (0)
Newfoundland	0	13	0	0	0
Total	17	271	50	7	25 (12)

^a^Number of patients whose isolates had *Burkholderia cepacia* epidemic strain marker (BCESM ) in the category “other genomovar III RAPD strain types”. Strain types 01, 02, 04, and 06 all encoded BCESM. RAPD = random amplified polymorphic DNA.

RAPD type 02 was the predominant genomovar III lineage in Canada. This is the same clone that is reported to have spread intercontinentally between Canada and the United Kingdom and is also known as ET12 (5). The *cblA* gene codes for production of a cable pilus thought to enhance adhesion to epithelial cells.

## Discussion

This analysis of isolates from the *B. cepacia* complex from a broad geographic distribution may facilitate insights into the epidemiology and virulence of this evolving class of bacteria in patients with CF. Most Canadian CF patients are cared for at centralized clinics in each province, and the data are relayed to a central registry at the national office of the Canadian CF Foundation. Regular audits by the Foundation enhance the quality of care at the individual CF clinics; as a result, microbiologic investigation of CF patient samples is optimized. The rate of misidentification of *B. cepacia* complex organisms in Canada has been very low since standard methods for culture and identification were publicized in 1997. Our recent experience contrasts with other reports of misidentification in Canada and the United States (25,26). An estimated 75% of prevalent *B. cepacia* infection was reported to the Canadian *B. cepacia* Complex Research and Referral Repository, and regional differences were similar to those recorded in the patient data registry. Therefore, the data reported here probably reflect true national trends in prevalence and strain distribution of *B. cepacia* complex organisms in CF patients in Canada.

Remarkable differences in prevalence and RAPD strain type clustering were noted among Canadian provinces. This was most striking in Ontario and Quebec, Canada’s two most populous provinces. These provinces are contiguous, but the prevalence of *B. cepacia* complex infection was about 10-fold higher in Ontario than in Quebec. Patients in these two provinces probably had very little contact with each other because of the geographically wide separation between clinics in the major population centers (Toronto and Montreal). Furthermore, the difference in primary language between the two provinces may have discouraged social mixing when opportunities arose. The predominant RAPD strain type recovered from patients in Ontario (RAPD type 02) was rarely cultured from patients in Quebec. This strain is prevalent throughout the United Kingdom and appears to have spread to Britain as well as to other parts of Canada as a result of common exposures in summer camps in Ontario (9).

Clustering of RAPD strain types by province suggests patient-to-patient spread. Most isolates of RAPD type 02 in British Columbia and in the Maritime provinces (Nova Scotia and Newfoundland) can be traced to care received in Toronto, where that is the predominant type (E. Tullis, pers. comm.). Since cohorting of patients was instituted in Canadian clinics in 1994, the spread of RAPD type 02 has slowed (24). Most new acquisitions of *B. cepacia* complex organisms in British Columbia since 1994 have been *B. multivorans* (24). Each new isolate has had a unique genetic fingerprint, suggesting that acquisition has not been from other patients, but from the environment. Recent reports from France and Italy describe the rhizosphere as an important environmental reservoir for *B. cepacia* complex isolates (27,28). We are searching for potential environmental reservoirs in Canada.

Clusters of common RAPD strain types in CF clinics have been from genomovar III. The four common RAPD strain types (01, 02, 04, and 06) all encode BCESM, but only type 02 encodes *cblA*. Although the latter appears to enhance adhesion of the bacteria to epithelial cells, the role of BCESM in transmissibility has not been determined.

Evidence of patient-to-patient spread of genomovar III *B. cepacia* complex strains has been documented in studies from different geographic regions (9,24). Our results support the likelihood that spread of these strains occurs throughout Canada. Most patients infected with genomovar III RAPD type 02 may have acquired the strain directly or indirectly from patients from Ontario. The factors that enhance such patient-to-patient spread have not been clearly determined, but segregation appears to have been successful in limiting transmission.

*B. multivorans* (genomovar II), in contrast to genomovar III, does not appear to have spread from patient to patient in Canada. Each of the isolates was typed by RAPD, and each had a unique genetic fingerprint. The only exceptions were isolates from a sibling pair who transiently shared the same strain. This observation contrasts with those from other parts of the world, where *B. multivorans* has been observed to cluster in CF clinics, suggesting patient-to-patient spread (12,29,30). The differences between our observations and those of others may be explained on the basis of difference in infecting strain types; the Canadian *B. multivorans* isolates may lack the putative factors necessary for patient-to-patient spread. Alternatively, infection control practices in Canada may differ from those elsewhere. The differences between the epidemiology of *B. multivorans* in Canada and the United Kingdom are analogous to that of *Pseudomonas aeruginosa* in CF. No evidence of patient-to-patient spread of *P. aeruginosa* in Canada has been documented (despite intensive investigation), but well-documented outbreaks of epidemic spread among patients in Liverpool and Manchester, United Kingdom (31,32), and Melbourne, Australia (33), have been reported.

The evidence of patient-to-patient spread of bacteria from the *B. cepacia* complex among patients with CF and the adverse prognosis of those who are infected (10,34) demands stringent efforts to prevent new acquisition. Strategies have been introduced in Canada, the United States, the United Kingdom, and elsewhere to limit spread both within and outside hospitals. These strategies appear to have limited the epidemic spread of certain clones of *B. cepacia* complex, but the prevalence of infection has remained largely unchanged. Infection control precautions are based on lessons learned from the control of spread of other respiratory tract pathogens; they may or may not be relevant for CF, with its unique host-pathogen relationship. Furthermore, *B. cepacia* complex is an opportunistic pathogen and is commonly found in the natural environment in such places as soil and plant roots (27,28,35–37). The mode of acquisition of *B. cepacia* complex in CF patients appears to be both from other patients and from the environment. Until more is known about risk factors for acquisition, rational infection control strategies will be difficult to design. We are attempting to identify the factors that may be correlated with acquisition of this problematic pathogen in Canadian patients with CF.
